# SUPPORTING RESIDENT ENGAGEMENT VIA WEB-BASED ASSESSMENT: EXPERIENCE OF ACTIVITY PROFESSIONALS IN LONG-TERM CARE

**DOI:** 10.1093/geroni/igac059.931

**Published:** 2022-12-20

**Authors:** Xiaoli Li, Cheng Yin

**Affiliations:** University of North Texas, Frisco, Texas, United States; University of North Texas, Corinth, Texas, United States

## Abstract

**Background:**

Resident engagement and activities have positive relationships with emotional wellbeing and physical health. However, regularly offered activities may not be suitable for all individuals and may be unsustainable when there are changes in residents’ health. Loneliness, depression, and social isolation are observed among residents in long-term care facilities. A web-based assessment system, “Elder Engagement Performance Improvement (EEPI),” has the potential to support resident engagement. Activity professionals are crucial in utilizing EEPI and engaging residents in meaningful activities, especially regarding their role in helping enhance resident engagement and wellbeing. This study aims explores activity professionals’ experiences in implementing the EEPI assessment. Method: Semi-structured interviews with 23 long-term care activity professionals (e.g., certified activities professionals, therapeutic recreation specialists, and activity assistants) were conducted and analyzed using qualitative content analysis.

**Results:**

Activity professionals` experiences showed the EEPI was a means in improving residents’ engagement quality and making engagement a more targeted outcome. Four main themes emerged: (a) Lack of assessment tools, (b) Acceptance of a web-based assessment system, (c) Barriers to using the EEPI (time arrangements, staffing issues, and being on the same page), and (d) Areas for job improvement (person-center care and measurable practices).

**Conclusions:**

The EEPI may serve as a valuable tool in long-term care facilities. The web-based assessment system facilitated activity professionals' daily work and involved the residents' input in their care, which may enhance resident engagement and reduce the risk of social isolation in long-term care facilities.

